# Research on Effects of the Thermal Stimulation by Moxibustion at Different Temperatures on Cardiac Function in Rats and on Mast Cells in the Local Site of Moxibustion

**DOI:** 10.1155/2013/545707

**Published:** 2013-07-21

**Authors:** Yao-Shuai Wang, Jian-Bin Zhang, Jin-Feng Jiang, Ling-Ling Wang

**Affiliations:** The Second Clinical College, Nanjing University of Chinese Medicine, Nanjing 210023, China

## Abstract

*Objective*. To observe effects of the thermal stimulation by moxibustion at different temperatures on cardiac function in brachycardia rat model and on mast cells in the local site of moxibustion at the Ximen Acupoint and to compare the differences of the effects of moxibustion at different temperatures. *Method*. Establish the brachycardia rat model with propranolol and observe effects of the thermal stimulation by moxibustion at different temperatures (38°C and 46°C). *Results*. The thermal stimulation by moxibustion at 2 temperatures may increase HR, MAP, LVSP, and +dp/dtmax and reduce t-dp/dtmax in brachycardia rats; the 46°C moxibustion group shows greater regulating effects on cardiac function in rats than that in the 38°C moxibustion group (*P* < 0.05). The thermal stimulation by moxibustion at 2 temperatures may promote degranulation of mast cells in the local site of moxibustion at the Ximen Acupoint; the degranulation rate in the 46°C moxibustion group is higher than that in the 38°C moxibustion group (*P* < 0.05). *Conclusion*. There is a certain association between the effect on the target organ and the effect in the local site of moxibustion. The moxibustion effect possibly resulted from local mast cells degranulation and different thermoreceptors activated by the thermal stimulation at different temperatures.

## 1. Introduction

As an important method in the traditional Chinese medicine, moxibustion is featured mainly with the “warm” advantage, by which it carries out stimulation at the Acupoint or the lesions and works by “stimulation from the outside and regulation from the inside”. The skin is the direct action site of moxibustion stimulation; selection of appropriate warm thermal stimulation to local skin, characteristics of responses to stimulation, and activation of relevant regulating mechanisms are based on the biological characteristics of skin, that is to say, to explore the effect law and action mechanism of moxibustion, attention has to be paid to the action characteristics of moxibustion and the characteristics of the action object of moxibustion. Modern neurobiological studies have demonstrated that [1] the body and skin tissues are distributed with protein receptors (transient receptor potential (TRP)) sensing different temperature ranges, which have their respective thresholds in sensing temperatures and may be activated by different warm thermal stimulation. The characteristics in the change of local temperatures, caused by moxibustion therapy, are greatly consistent with categories and distribution characteristics of relevant temperature-sensing subtypes of TRP, such as the TRPV1 in the TRP family which may be activated by thermal stimulation (>43°C) [2]. Recent studies have found that TRPV1 expression and activation of skin cutin cells may induce inflammatory reaction [3]. Activated TRPV1 can make mast cell degranulation release proinflammatory and itch causing media to participate in the inflammatory reaction [4]. The inflammatory reaction is associated with pathological physiological function in the cardiac arrhythmias [5]. Moreover, TRPV1 has an important regulating effect on the cardiovascular system [6]. Moxibustion can effectively regulate blood, blood flow and microcirculation condition, and diastolic function [7, 8]. Studies have shown effects of acu-moxibustion associated with mast cells in acupoints [9]. From the previously mentioned, attention needs to be paid to what effects may be produced by the thermal stimulation at two temperatures below and above 43°C on cardiac function in rates, as well as on skin in the local site of moxibustion, whether there is any difference or association of effects, and whether they may provide any basis for optimizing the clinical operating program of moxibustion.

## 2. Materials and Methods

### 2.1. Materials

#### 2.1.1. Animals and Grouping

Healthy male SD rats of clean grade, with body weight of 280–380 g, Certificate no. SCXK (Shanghai) 2007-0005, were purchased from Shanghai Slac Laboratory Animal Co., Ltd. and bred in batches. The experiment was carried out in 1 week after the rats were accommodated. The rats were divided into the following groups by the random number table: Model Control Group, Moxibustion Group 1, and Moxibustion Group 2. The number of rats in each group would support to obtain 8 complete data successfully. During the experiment, the animal treatment measures were in strict accordance with the regulations in the *Instructive Opinions on Humanitarian Treatment of Laboratory Animals*, issued in 2006 by the Ministry of Science and Technology of the People's Republic of China and the provisions for care and use of laboratory animals by Nanjing University of Traditional Chinese Medicine.

#### 2.1.2. Main Laboratory Equipment

Moxa strips for animals (in the diameter of 7 mm, Nanyang Chinese Medicine Moxa Company), thermostatic operating table for animals (ST-1 model, Chengdu Instrument Factory), multichannel physiological signal acquisition system (RM 6240 model, Chengdu Instrument Factory), temperature measuring device (HC-04 model, Hangzhou Hongchang Technology Company), microsyringe pump (ALC-IP800, Shanghai Alcott Biotech Co., Ltd.), full automatic tissue processor (TP1020, German LEICA Company), paraffin slicing machine (RM2145, German LEICA Company), optical microscope (BX60, Japanese OLYMPUS Company), and video camera (DP71 Image Acquisition System, Japanese OLYMPUS Company) were used.

### 2.2. Methods

#### 2.2.1. Electrophysiological Experimental Method

**Figure 1:**
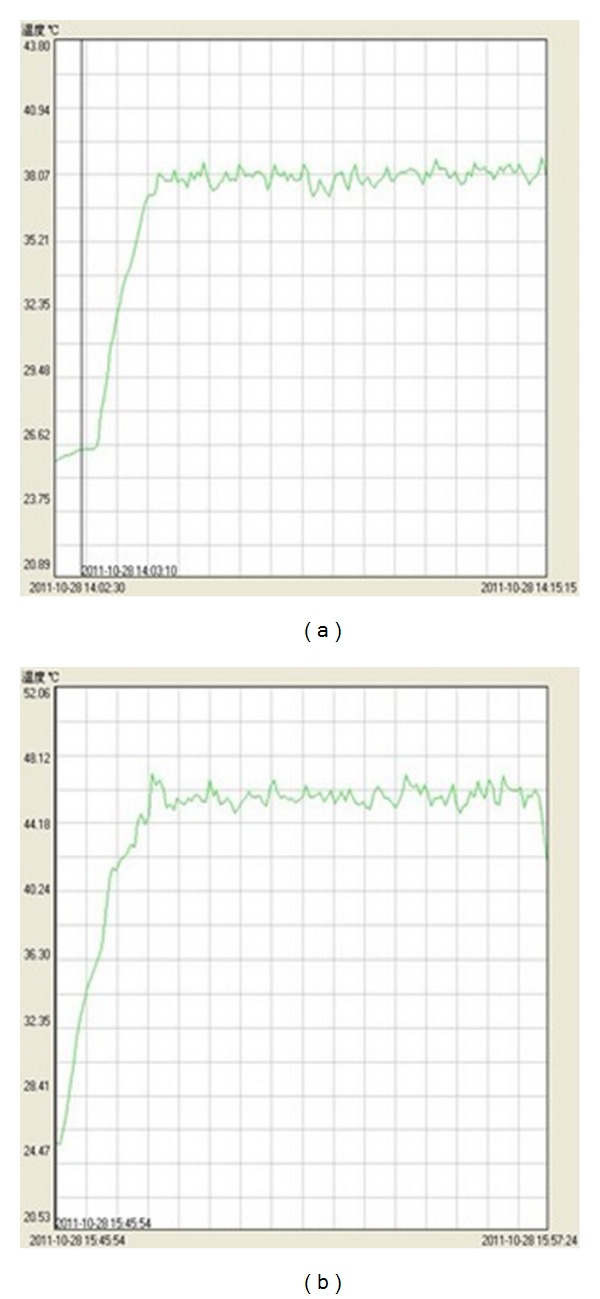
Sample curve picture of the point skin temperature during hot stimulation process of moxibustion group, Moxibustion Group 1 (a), Moxibustion Group 2 (b).

Fast a male SD rat overnight for 12 hours, with free access to water, weigh the rat, carry out anesthesia of the rat with 20% urethane (ethyl carbamate) in the dose of 1 g/kg body weight, fix the rat in the thermostatic operating table for animals, and connect with the electrocardiograph and the temperature transducer (via the anus of the rat); once the electrocardiogram and body temperature of the rat are stable (37°C), carry out intubation in the left ventricle, as well as in the femoral artery and femoral vein of the right lower limb, to measure left ventricular pressure and arterial pressure, and administer the drug intravenously. Model control group 10 minutes after the operation, if the measurement indexes are stable, start recording data; 5 minutes later, rapid inject 0.4% inderal (4 mg/kg) via the femoral vein; then slowly inject intravenously to maintain inderal at the dose of 0.25 mg/kg/min with the microsyringe pump for 60 min, to establish the bradycardia model; no any other interventions are carried out. Moxibustion group 1: establish the model as described in the model control group. At 20 min during administration of inderal at the maintenance dose, ignite the customized slender moxa strip (20 cm long and in the diameter of 7 mm), carry out moxibustion directly to stimulate at the Ximen(PC4) Acupoint in the left upper limb of the rat for 10 min, place the probe of the temperature controller at the surface of the skin at the acupoint for moxibustion to monitor real-time temperature, and control the temperatures in the local skin for moxibustion at 38°C ± 1°C (Figure 1(a)); the flaming moxa strip and skin are 35 ± 5 mm apart; after moxibustion, continue observation for another 30 min. Moxibustion group 2: establish the model as described in the model control group. At 20 min during administration of inderal in the maintenance dose, carry out moxibustion for thermal stimulation of 10 min at 46°C, carry out the intervention similar to that as described in moxibustion group 1; during thermal stimulation by moxibustion, control the temperature in the local skin for moxibustion at 46°C ± 1°C Figure 1(b), and the flaming moxa strip and skin are 12 ± 2 mm apart; after moxibustion, continue observation for another 30 min. During moxibustion, gently tap any moxa ashes at the tip of the moxa strip to the ashtray, based on the combustion state of the moxa strip, to always maintain cleanliness of the operating interface. The Ximen Acupoint in the left limb is selected; the positioning method of the acupoint is the anthropomorphic comparative method, that is, Ximen: In the inner side at the 1/2 folding point of the forearm, and in the middle of the gap between the radius and the ulna. The reason for the choice of the temperature is that, based on our observations, less than 38°C thermal stimulus, the human body for the warm feeling is not obvious, and above 46°C thermal stimulus, the human body for the hot feeling is very obvious, difficult to continue to endure. During the whole course, using RM6240B multichannel physiology recorder (parameters: acquisition frequency 1 kz, scan speed 40 ms/div, and filter frequency 30 Hz) to simultaneously monitor and record many indexes for cardiac function and hemodynamics, including heart rate (/min), mean femoral artery pressure (mmHg), left ventricular systolic pressure (mmHg), maximum rate of left ventricular pressure rise (mmHg/s), and time (ms) from the beginning of left ventricular systole to the maximum rate of left ventricular pressure rate, and so forth.

#### 2.2.2. Morphological Experimental Method

*(1) Obtain Materials*. After completion of the monitoring and recording of the electrophysiology experiment, sacrifice the rats and obtain materials. Rapidly extract skin tissues in the local site at the Ximen Acupoint in the rats of each group with the ophthalmic forceps and the surgical scissors, and place into 10% formaldehyde solution to store and fix for 1 week. 

*(2) Processing prior to Staining*. (1) Trimming: take out the skin tissues that are sheared and then place them in 10% formaldehyde solution and fix them for 1 week, trim the tissues with the surgical knife to a size of approximately 0.7 cm ∗ 0.7 cm ∗ 0.3 cm, and then place them into 70% alcohol to remove any residual formaldehyde solution in the tissues. (2) Dehydration: place the trimmed tissues into the full automatic tissue processor for dehydration. (3) Embedding: carry out paraffin embedding of the processed tissues. (4) Slicing: carry out common slicing by the slicing machine to obtain slices in the thickness of 5-6 *μ*m, and place them into hot water to be flattened. Then slowly take out the slices, rapidly shake off water, and then slowly place them on the well-labeled glass slides, to prevent any air bubbles formed between tissue and slide; in case of any air bubbles, prick off carefully with the acupuncture needle. Bake on a slice-baking plate until the paraffin turns transparent, and place into the slice-baking oven, bake overnight at 60–64°C, and then allow to cool for use.

*(3) Toluidine Blue Staining*. (1) Commonly remove paraffin from slices, and then wash with water; (2) dip-dye with 0.5 toluidine blue solution (to 0.5 g of toluidine blue dye, add water to the volume of 100 mL); (3) wash with water, to remove any excessive staining solution; (4) carry out differentiation with the differentiation solution (mix 0.5 mL of glacial acetic acid and 99.5 mL of distilled water and shake well); (5) wash with water and air-dry; (6) carry out dehydration gradiently with alcohol; (7) treat with xylene until transparent; and (8) carry out fixation of slices with neutral balsam.

#### 2.2.3. Statistical Method

The data are expressed as mean ± standard deviation $\left(\overset{-}{X}\pm S\right)$ and are analyzed and processed by the SPSS17.0 statistical software. The data obtained from each group are subject first to the homogeneity test of variance. In case of homogeneity of variance, the one-factor analysis of variance is used; if there is difference of population means, the pair-wise comparisons are carried out by the LSD test (in case of heterogeneity of variance, the pair-wise comparisons are carried out by Brown-Forsythe approximate analysis of variance and Games-Howell method); *t* test is used for comparison between two independent samples. It is regarded as significant in case of *P* < 0.05. 

## 3. Results

During the experiment, all deaths due to anesthesia, operative accidents, or drug administration, and so forth, as well as all abnormal responses to intervention of the established model, are not included for observation; 8 valid experimental cases were obtained in each group, so a total of 24 cases were obtained.

### 3.1. Results from Electrophysiological Experiment

Statistical analysis was carried out by the 5 min means for each of the indexes collected, such as HR, MAP, and cardiac function (LVSP, +dp/dtmax, and t-dp/dtmax). To reduce any statistical errors due to individual differences, the changes before and after intervention in each experimental animal were expressed in percent: Change Rate (%) = (value after intervention − value before intervention)/value before intervention × 100%. The results were provided as follows.

#### 3.1.1. Characteristics in the Changes of HR and MAP in the Brachycardia Rat Models Induced by Inderal

The administration of inderal resulted in rapid decrease of HR and MAP in rats (on average, in 60 minutes, HR was decreased by 34.98% ± 5.86%, and MAP was decreased by 33.21% ± 5.63%). In 20 minutes after administration of inderal, the model was stable successfully; during 40-min maintenance after 20 min administration of inderal, the mean change rate of HR was −0.16% ± 1.98% and that of MAP was 0.37% ± 2.09%. The results showed that the method was able to establish brachycardia rat models with stable effects (Table 1).

#### 3.1.2. Waveform Changes of Left Ventricular Pressure and Arterial Pressure before and after Model Establishment and Intervention by Moxibustion

**Figure 2:**
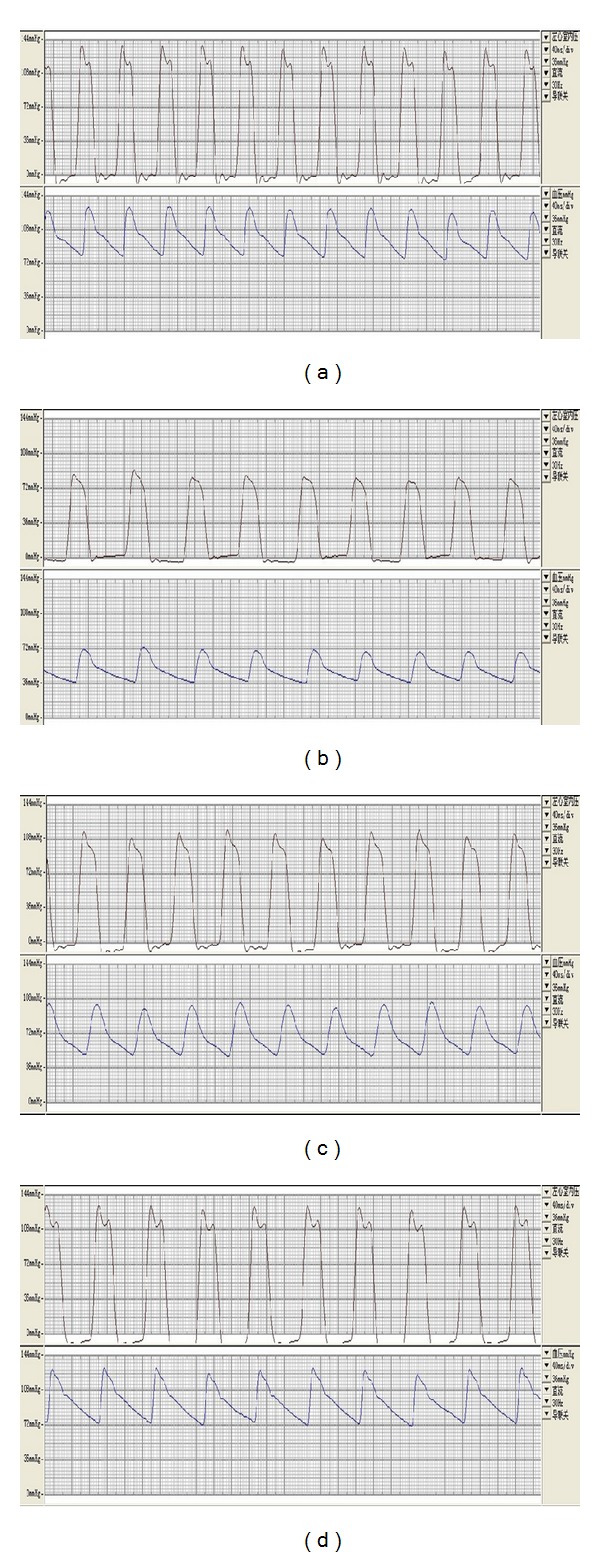
Sample waveshape picture of the LVP, AP of each group: normal condition (a), the model was established successfully with inderal (b), after thermal stimulation at 38°C (c), after thermal stimulation at 46°C (d).

After the model was established successfully with inderal, the waveforms of left ventricular pressure and arterial pressure were obviously decreased than normal; after thermal stimulation by moxibustion at 38°C and 46°C, the waveforms of left ventricular pressure and arterial pressure were recovered to some extents than those in the model (Figure 2).

#### 3.1.3. Intergroup Comparisons of Mean Change Rates of Each of the Indexes, Such As HR, MAP, LVSP, +dp/dtmax, and t-dp/dtmax, after Intervention by Moxibustion

Compared with those in the model control group, the thermal stimulation in the Moxibustion group 1 was able to increase HR, MAP, LVSP, and +dp/dtmax and reduce t-dp/dtmax in rats; however, the effects were not significant (*P* > 0.05). Compared with those in the model control group, the thermal stimulation in the Moxibustion group 2 was able to increase HR, MAP, LVSP, and +dp/dtmax and reduce t-dp/dtmax in rats, and the effects were significant (*P* < 0.05); moreover, the effects of the thermal stimulation in the moxibustion group 2 were higher than those in the moxibustion group 1 (*P* < 0.05) (Table 2).

### 3.2. Results from the Morphological Experiment

The morphology of mast cells was observed under the 10 × 40 optical microscope, and mast cells with degranulation in skin were counted. 

#### 3.2.1. Results of Morphological Observations of Mast Cells

**Figure 3:**
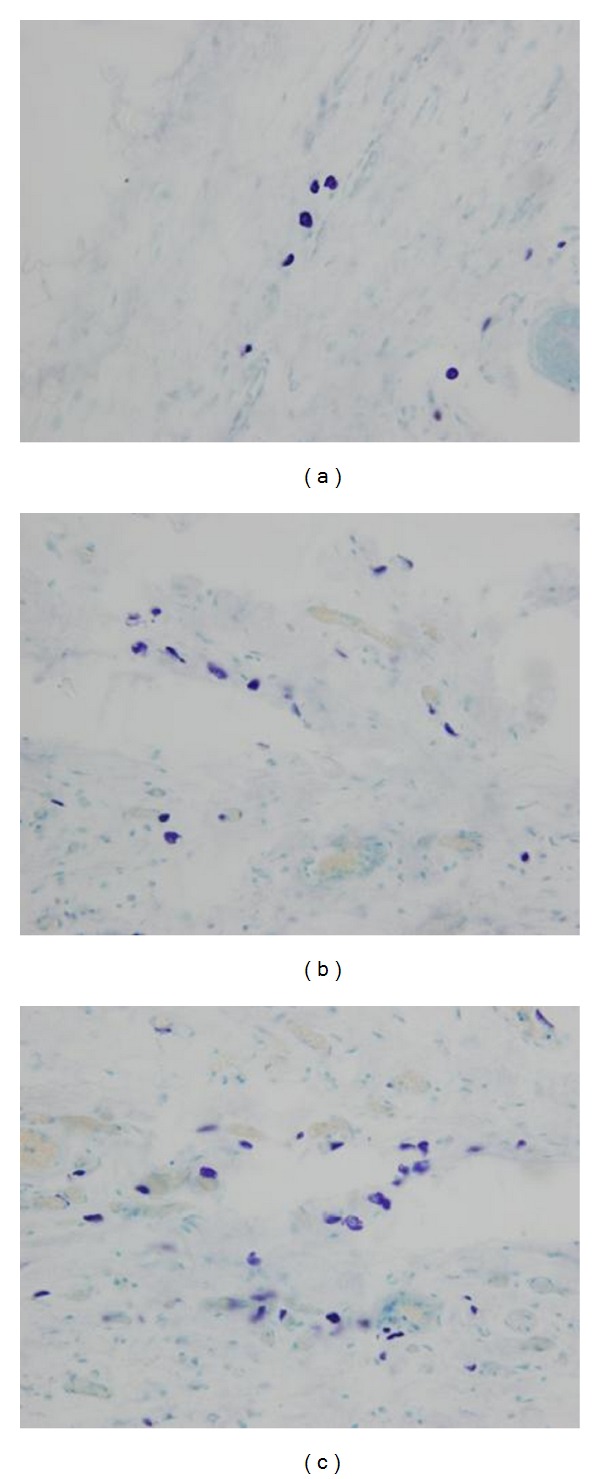
Morpohologies of Mast cells in the Local Site of the Ximen Acupoint: Model Control Group (a), Moxibustion Group 1 (b), Moxibustion Group 2 (c).

Findings of mast cells in slices of skin tissues in the area of the Ximen Acupoint were observed under optical microscope. (1) Model control group: the mast cells were of smooth cell walls and of complete morphology (Figure 3(a)); (2) moxibustion group 1: the mast cells in the local site were of incomplete morphology and were with ruptured cell membranes (Figure 3(b)); (3) moxibustion group 2: the mast cells in the local site were with ruptured cell membranes, and a large amount of granules were scattered around the cell body, and there were significant degranulations (Figure 3(c)).

#### 3.2.2. Degranulation Rate of Mast Cells

The criteria for counting mast cells with degranulation were: count as 1 when the mast cell was with ruptured cell wall, and there were scattered granules in the matrix. Degranulation rate = count of mast cells with degranulation/total count of mast cells × 100%. The mean of degranulation rates from 4 slices which were closest to the local site of moxibustion was calculated in each tissue sample, add the mean of degranulation rates of this group all tissue samples together, and divided by the number of samples in each group, then the degranulation rate of mast cells could be calculated.

If no intervention was carried out to the skin at the Ximen Acupoint in rates, the degranulation rate in the local site was as lowas 12.9%; after thermal stimulation bymoxibustion, the degranulation rate of mast cells was significantly increased (*P* < 0.05) (Table 3).

## 4. Discussion

From ancient times, moxibustion is one of the important methods in treatment of cardiovascular diseases in clinical practices. It is a crucial problem for moxibustion research how to objectively assess the effects of moxibustion on heart and blood vessels, to explore the possible mechanism for differences of effects, resulted from stimulation by moxibustion at different temperatures as an influencing factor, and to provide a basis for optimizing the clinical operating program of moxibustion. In this experiment, propranolol, a *β*-receptor blocker, was used to establish the acute brachycardia animal model [10, 11]. It is an easy and simple technique, with good repeatability. The narcotic agent, urethane, is of wide safety margin, with an easily controlled dose, and has small toxicity to heart. The cardiovascular rat model is of good stability, and moxibustion at the Ximen Acupoint has good effects against arrhythmia; they have been verified well in the previous studies of the research group [12, 13]. In this experiment, the indexes including HR, MAP, LVSP, +dp/dtmax, and t-dp/dtmax were analyzed comprehensively; in this way, it may completely reflect the effects of the thermal stimulation by moxibustion on myocardial contractility and peripheral circulation center function. HR may reflect change in the myocardial contractility visually and clearly. After opening of the aortic valve, the left ventricle ejects blood to the artery. The stronger the left ventricle contracts, the more blood it ejectss and then the higher the blood pressure is; the afterload resisting ejection of the left ventricle is mainly the peripheral blood pressure (the mean femoral artery pressure was mainly observed in this experiment). The left ventricular systolic function is associated with the afterload of the left ventricle. MAP may reflect the change of the peripheral circulation center function. +dp/dtmax is very sensitive to interventions by various variable stresses and, to a certain extent, is influenced by and positively correlated with heart rate and the preload and afterload; the increase of +dp/dtmax with concurrent reduction of t-dp/dtmax suggests increase of myocardial contractility. The results of this study found that the thermal stimulation by moxibustion at 2 temperatures (38°C and 46°C) may promote increase of HR and MAP in propranolol-induced brachycardia model rats, recovery of LVSP and +dp/dtmax, and reduction of t-dp/dtmax, suggesting that the thermal stimulation by moxibustion at 2 temperatures (38°C and 46°C) has the effects of improving cardiac function in brachycardia rats, and the effects of the thermal stimulation by moxibustion at 46°C are obviously stronger than those of the thermal stimulation by moxibustion at 38°C. Our results are in conformity with ancient doctors' point of view that enough strength of thermal stimulation achieve good curative effect.

The ancient doctors emphasis moxibustion heat stimulates the skin local inflammatory response is beneficial to the treatment of diseases. Our study demonstrated that the thermal stimulation by moxibustion may influence the morphology of mast cells in the local site of moxibustion, promote their degranulation, and similarly, the thermal stimulation by moxibustion at 46°C is obviously stronger than that by moxibustion at 38°C. The results suggested that higher or lower mast cell degranulation rate influenced the improvement of cardiac function, so the characteristics of warm thermal stimulation by moxibustion are certainly associated with the effects on the target organ, as well as on the local site of moxibustion. The local site of moxibustion is the direct object of moxibustion action, and is one of the key links for the starting mechanism of the moxibustion effects. The therapeutic effect of moxibustion is to regulate the body by stimulating the acupoints, while the regulating effects are dependent on receptors in the acupoint area. Recent studies have believed that the skin is an important neuroendocrine organ, which may synthesize and secrete many neurotransmitters and peptide substances [14], while the TRP channel is probably the significant potential molecular target of the warm thermal effects by moxibustion. The TRP channel is widely distributed in mast cells in the body [15], mainly including TRPV1 and TRPV2, which opening effects may directly influence the function and efficacy of mast cells. Especially, TRPV1 calcium ion channels of mast cells may participate in regulating mast cell biological functions and the pathological process [16]. Some scholars proposed the “axonal reflex-linkage hypothesis” [17], in which the synaptic connection between mast cells and nerve fiber acts as a relay baton and the information is transmitted in the form of stimulating mast cells to degranulate and release active substances and then to activate adjacent nerve terminals. Galli proposed “Mast cells cytokine cascade reaction” [18], based on the characteristics of Mast cells secreting many transmitters and cytokines. Therefore, we deduced that different moxibustion methods may produce different characteristics of temperature changes, activate different TRP subtypes, induce different biological effects, and finally achieve different therapeutic effects.

In this study, the relevant new biological knowledge of the skin was used as the basis for this study, to focus on differences of the effects due to warm thermal stimulation by moxibustion at different temperatures and to explore their association with physiological feedback responses from different thermoreceptors in the body. This was because that, during the previous research on the action mechanism of moxibustion, we paid more attention to the effects of moxibustion therapy on the diseased target organ and overlooked the unique path and mechanism of starting effects by moxibustion on the target organ. Every effective therapeutic measure may produce effects on the target organ, and the specificity of its action path is the important basis for existence of the method. Based on the stimulation-response process of thermal stimulation by moxibustion → local reception → temperature → chemical conjugation linkage → biological information cascade reaction → effect realization [19], we believed that, due to different temperatures of thermal stimulation, there are differences of excitation receptors; the thermal stimulation at 46°C may activate TRPV1 in the local site of moxibustion and promote degranulation of Mast cells, and the consequent effects may participate in regulation of cardiac function; therefore, the thermal stimulation at 46°C has better improvement effects on cardiac function in brachycardia rats. The thermal stimulation and the resulting inflammatory reaction are the most basic features of moxibustion effect [20], so in our study, attention was paid to the characteristics of the effects in the local site of moxibustion; it may help in further research of the paths of neurology, endocrine, and immune network interaction of moxibustion and also provide a new path to clarify the action mechanism of the effects of moxibustion. 

## Figures and Tables

**Table 1:** Mean change percentage of the model group HR, MAP (%).

Model group	*N*	HR	MAP
60 minutes after administration of inderal	8	34.98 ± 5.86	33.21 ± 5.63
After 20-min administration of inderal	8	−0.16 ± 1.98	0.37 ± 2.09

**Table 2:** Mean change percentage of the HR, MAP, LVSP, +dp/dtmax, and t-dp/dtmax of different groups after moxibustion treatment.

Group	*N*	HR (/min)	MAP (mmHg)	LVSP (mmHg)	+dp/dtmax (mmHg/s)	t-dp/dtmax (ms)
Model control group	8	−0.023 ± 0.099	−0.033 ± 0.086	0.045 ± 0.093	0.057 ± 0.14	0.018 ± 0.113
Moxibustion group 1	8	0.044 ± 0.212	0.063 ± 0.078	0.087 ± 0.03	0.14 ± 0.052	−0.026 ± 0.04
Moxibustion group 2	8	0.143 ± 0.074^∗▲^	0.425 ± 0.284^∗▲^	0.255 ± 0.109^∗▲^	0.358 ± 0.143^∗▲^	−0.19 ± 0.16^∗▲^
Analysis of variance						
*F*		10.660	14.963	13.875	13.568	7.217
*P*		0.001	0.000	0.000	0.000	0.004

Note: the values in the table for each index value variation rate.

Compared with model control group *P < 0.05.

Compared with moxibustion group 1 ^▲^ *P* < 0.05.

**Table 3:** Degranulation rate of mast cells.

Group	*N*	Degranulation rate of mast cells %
Model control group	8	12.9 ± 2.8
Moxibustion group 1	8	37.6 ± 5.9*
Moxibustion group 2	8	68.5 ± 6.3^∗▲^

Note: compared with model control group *P < 0.05.

compared with moxibustion group 1 ^▲^ *P* < 0.05.

## Abbreviations

AP: Arterial pressure
+dp/dtmax: Maximum rate of left ventricular pressure rise
HR: Heart rate
LVSP: Left ventricular systolic pressure
MAP: Mean arterial pressure
MCs: Mast cells
TRP: Transient receptor potential
TRPV1: Transient receptor potential vanilloid subfamily member 1
t-dp/dtmax: Time from the beginning of left ventricular systole to the maximum rate of left ventricular pressure rate.
